# Enhanced Multiscale Human Brain Imaging by Semi-supervised Digital Staining and Serial Sectioning Optical Coherence Tomography

**DOI:** 10.21203/rs.3.rs-4014687/v1

**Published:** 2024-03-21

**Authors:** Shiyi Cheng, Shuaibin Chang, Yunzhe Li, Anna Novoseltseva, Sunni Lin, Yicun Wu, Jiahui Zhu, Ann C. McKee, Douglas L. Rosene, Hui Wang, Irving J. Bigio, David A. Boas, Lei Tian

**Affiliations:** 1 Department of Electrical and Computer Engineering, Boston University, 8 St Mary’s St, Boston, MA, 02215, USA.; 2 Department of Electrical Engineering and Computer Sciences, University of California, Cory Hall, Berkeley, California, 94720, USA.; 3 Department of Biomedical Engineering, Boston University, 44 Cummington Mall, Boston MA, 02215, USA.; 4 Department of Computer Science, Boston University, 665 Commonwealth Ave, Boston, MA, 02215, USA.; 5 Boston University Alzheimer’s Disease Research Center and CTE Center, Boston University, Chobanian and Avedisian School of Medicine, Boston, MA, 02118, USA.; 6 Department of Neurology, Boston University, Chobanian and Avedisian School of Medicine, Boston, MA, 02118, USA.; 7 VA Boston Healthcare System, U.S. Department of Veteran Affairs, Jamaica Plain, MA, 02130, USA.; 8 Department of Psychiatry and Ophthalmology, Boston University School of Medicine, Boston, MA, 02118, USA.; 9 Department of Pathology and Laboratory Medicine, Boston University School of Medicine, Boston, MA, 02118, USA.; 10 Department of Anatomy & Neurobiology, Boston University Chobanian & Avedisian School of Medicine, Boston, Massachusetts, USA.; 11 Athinoula A. Martinos Center for Biomedical Imaging, Department of Radiology, Massachusetts General Hospital/Harvard Medical School, Charlestown, MA, 02129, USA; 12 Neurophotonics Center, Boston University, Boston, MA, 02215, USA.

## Abstract

A major challenge in neuroscience is to visualize the structure of the human brain at different scales. Traditional histology reveals micro- and meso-scale brain features, but suffers from staining variability, tissue damage and distortion that impedes accurate 3D reconstructions. Here, we present a new 3D imaging framework that combines serial sectioning optical coherence tomography (S-OCT) with a deep-learning digital staining (DS) model. We develop a novel semi-supervised learning technique to facilitate DS model training on weakly paired images. The DS model performs translation from S-OCT to Gallyas silver staining. We demonstrate DS on various human cerebral cortex samples with consistent staining quality. Additionally, we show that DS enhances contrast across cortical layer boundaries. Furthermore, we showcase geometry-preserving 3D DS on cubic-centimeter tissue blocks and visualization of meso-scale vessel networks in the white matter. We believe that our technique offers the potential for high-throughput, multiscale imaging of brain tissues and may facilitate studies of brain structures.

## Introduction

The human brain consists of an estimated 86 billion neurons (1), which form intricate connections and networks that underlie the complex functions. To gain new insights into the brain, major efforts have recently been made to develop multiscale imaging technologies for visualizing anatomical structures with microscopic resolution across cubic centimeters of tissue. The most widely used techniques for visualizing anatomical and neuronal structures are based on histological staining. Gallyas silver staining is used to characterize myelin content and neuronal structures, as well as to identify pathological features of neurodegenerative diseases in human brain tissue (2, 3). To create a high-resolution 3D model of the cytoarchitecture, the BigBrain project (4) reconstructed a whole human brain with more than 7000 histological sections, which involves slicing the tissue into 20-μm sections, staining with silver halide to reveal cellular and fiber structures, and registering the slices in 3D. However, these histological staining processes are generally complex, labor-intensive, time-consuming, and prone to experimental error and staining variability. Furthermore, the slicing, mounting, dehydration, and staining inevitably cause tissue damage and slice-specific distortions, which can limit the accuracy of 3D alignment and reconstruction of structures at the micron scale (5, 6). Therefore, there is a growing need for developing 3D pathology imaging techniques, especially label-free techniques that can provide high-resolution 3D visualizations of brain tissues with minimal tissue damage and distortion, and that can reduce the need for physical staining (PS) (7–10).

Optical coherence tomography (OCT) is a label-free imaging technique that allows high-resolution 3D visualization and quantification of intrinsic optical properties of tissue, such as the scattering coefficient and back-scattering coefficient (11, 12). Recently, OCT has shown great promise in brain imaging applications, such as visualizing single neurons (13), fiber tracts (14), and the laminar structure of the cerebral cortex in the human brain (15, 16). While traditionally limited by light penetration, serial sectioning OCT (S-OCT) integrates OCT with a vibratome slicer to enable 3D imaging of cubic centimeters of tissue (17). S-OCT permits straightforward and accurate 3D high-resolution reconstruction of large-scale brain anatomy, microstructures, and tractography (17–19) with minimal tissue distortion. This is achieved through the use of a serial imaging protocol (20), where OCT imaging of the top ~150 μm thick tissue is alternated with the slicing off of the superficial tissue, thus reducing cutting-induced distortion after imaging. This enables accurate reconstruction of the complex 3D structures of brain tissues without requiring sophisticated inter-slice registration algorithms. Despite its ability to routinely generate large-scale volumetric brain imaging data, S-OCT still requires considerable expertise to identify and annotate anatomical and neuronal features for further analysis (11, 14, 17, 21). Our goal is to augment S-OCT with a digital staining (DS) technique that enables straightforward 3D histology on large-scale human brain tissues.

In the past few years, deep learning methods have revolutionized the field of DS, which aims to transform label-free images into histological staining-like images using a computational model (22). DS offers a fast and low-cost alternative to conventional PS methods. Several DS models have been developed that transform different pairs of input-output imaging modalities. However, most existing DS methods rely on supervised learning methods, which requires paired images of the tissue slice with and without staining for model training. To ensure accurate DS results, cross-modal registration between the image pairs with pixel-level accuracy is crucial (23–26). However, obtaining such image pairs is difficult and often involves sophisticated image registration procedures (22, 24). To overcome this challenge, some recent studies have explored unsupervised image translation models for DS, which only need unpaired collections of images from the two modalities for model training (8, 27–30). The most popular unsupervised method is CycleGAN (31), which comprises two sets of generators and discriminators that enforce cycle consistency and content preservation for the image translation task. A recent improvement over CycleGAN is Contrastive Unpaired Translation (CUT) (32), which uses contrastive learning to achieve better structural and content preservation with only one set of generator and discriminator, and has demonstrated superior performance in DS tasks (28). However, these unsupervised models still lag behind supervised models in terms of accuracy (22).

Here we present a new *semi-supervised* learning framework for DS using a *limited amount* of *weakly paired* image data. As a proof-of-concept demonstration, we use our DS model to translate S-OCT images to Gallyas silver staining. Our DS model consists of two novel modules that address several challenges in our technique. Our main model is based on the CUT framework to perform DS using unpaired training data. This module combines contrastive learning and adversarial learning to address the lack of paired imaging data since the physically stained images were obtained from unordered adjacent brain tissue sections to the OCT-imaged sections and were confounded by tissue damage and distortion during the staining process.

To improve the accuracy of the unsupervised model, we augment it with semi-supervision from two auxiliary tasks. Firstly, we devise a pseudo-supervised learning module by training the DS network on a pseudo-paired training dataset that is generated using our previously established biophysical model. Our previous work has revealed a linear correlation between the OCT scattering coefficients (SC) and the optical density (OD) computed from the Gallyas silver stained image (21). Based on this similarity prior, this module learns to translate the generated OD back to the Gallyas silver stain, acting as a proxy supervision for learning the translation from OCT-SC to Gallyas silver stain. This naturally pixel-aligned pseudo supervision augments the training data, enabling training the DS model effectively despite the limited data available to our task due to the scarcity of the human brain samples. Additionally, when combined with the adversarial learning component in the CUT backbone, the domain gap between the OCT-SC images and OD maps are effectively mitigated by the mechanism of domain-adversarial training (33). Secondly, we develop an unsupervised cross-modality image registration module that aligns the adjacent Gallyas image with the OCT-SC image. This module enables the DS model to utilize the geometric similarity information provided by the adjacent slices, thereby guiding the image translation process. To train the registration network effectively, we introduce a novel two-stage, multiscale training strategy. It allows the network to learn image registration at the “global” whole slide image (WSI) scale, while simultaneously learning image translation at the “local” image patch scale. Furthermore, this novel training strategy facilitates collaborative training between the DS model and the registration model, leading to more effective enforcement of high-quality DS results.

**Fig. 1.**
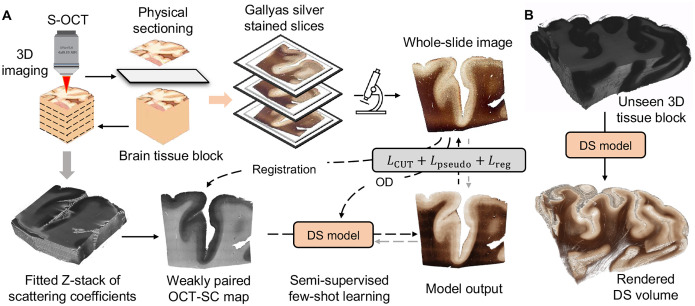
Overview of the proposed OCT DS technique. (**A**) Data acquisition and DS model. S-OCT alternates between 3D imaging and tissue sectioning to acquire a stack of block-face OCT images, which are then processed to compute the scattering coefficient (OCT-SC) map stack. Sectioned sample slices are physically stained and imaged. The DS neural network is trained from a few weakly-aligned pairs of OCT-SC and Gallyas silver-stained images. (**B**) After the DS model is trained, it can perform inference on completely new slices of OCT-SC images for volumetric DS.

We present our DS pipeline for data acquisition and deep learning model training in Fig. 1A. We use S-OCT to obtain label-free volumetric data of human brain samples. We then process the OCT data to calculate the SC maps (11) (see details in Methods). Next, we develop a deep learning DS model that transforms OCT-SC images into Gallyas silver stain images. We choose OCT-SC as the input for the DS model instead of the raw OCT measurements because SC measures the intrinsic optical properties of the tissue and eliminates the inhomogeneity in the raw OCT intensity by using a nonlinear model-fitting process (11). Moreover, a biophysical model from our previous work showed that OCT-SC mainly depends on the contribution of myelin content, which is captured by the OD of the Gallyas silver staining (21). We hypothesize that the correlation between these two modalities can be leveraged to create a more accurate image-to-image mapping using a deep learning model. During S-OCT, we also collect a few unordered tissue slices that are physically stained for DS model training and evaluation. The deep learning model is trained on a few weakly-aligned pairs of OCT-SC and Gallyas silver stained WSIs. The inference stage of the DS model is shown in Fig. 1B. After the model is trained, it can be applied on any OCT-SC maps to enable 3D neurohistology on cubic centimeters of brain tissue and visualize mesoscopic brain structures.

First, we present the OCT DS results on single-section tissues from various cerebral cortex samples and compare them with PS results from adjacent sections. We demonstrate that DS exploits the quantitative nature of OCT-SC and thus can produce consistent staining quality across different samples. Compared to PS, DS reveals comparable mesoscopic (~10 μm) structures in different tissue regions without introducing staining variability across samples and experiments. In addition, we show that DS enhances contrast across cortical layer boundaries and can consistently differentiate cortical layers IV, V and VI. Next, we show a 3D-rendered volumetric DS result on a cubic centimeter-scale tissue block that was not used for training the DS model. The result shows geometry-preserving 3D staining on large-scale brain tissue and visualization of vessel structure in the white matter region. Finally, we showcase a pilot study on the generalization performance of our method - we apply the DS model trained on cortex regions to samples from other anatomical regions acquired from different OCT setups.

In summary, we present a novel deep learning technique for DS of OCT images for large-scale human brain imaging. Our method allows direct visualization of important mesoscopic 3D brain features, including myeloarchitecture of the cerebral cortex and main 3D blood vessel network in the white matter, with contrast that closely resembles Gallyas-silver staining. Our method has several advantages over traditional PS, such as reducing staining variability, preserving complex brain 3D geometry and facilitating volume generation across cubic centimeters of tissue. Our method also improves the interpretability of the label-free OCT modality for brain imaging. However, our method also faces some limitations that originated from our current S-OCT system, such as artifacts from image stitching (12, 14), uneven tissue sectioning, speckle noise, and limited lateral and axial resolution due to the SC model fitting. Although our technique is sensitive to fiber structures in the gray matter, the speckle noise and limited resolution resulted in discontinuities and grainy artifacts in the DS results. We expect that these issues will likely be overcome by future generations of high-resolution S-OCT systems (34, 35) and improved processing algorithms. Despite current limitations, we believe that our semi-supervised learning-based DS framework is broadly useful to other bioimaging modalities and DS applications. Furthermore, our work has significant implications for quantitative volumetric neuropathology. The integration of DS techniques with S-OCT has great potential for high-throughput, multiscale human brain imaging. The data generated from this technique could help better understand the meso- and micro-structure of brain tissues and their role in disease development, and ultimately enhance our knowledge of the brain’s structure and function.

## Results

### Digital staining by semi-supervised learning using weakly-paired images

We formulate the DS task as a weakly-paired image translation problem because we do not have access to pixel-aligned image pairs of OCT-SC and PS images. To achieve better performance than fully unsupervised methods, we exploit the side information provided by the structural and content similarity between the adjacent sections in the imaging data, as well as a biophysical model for linking OCT-SC and the contrast in Gallyas silver stain in a semi-supervised deep learning framework.

**Fig. 2.**
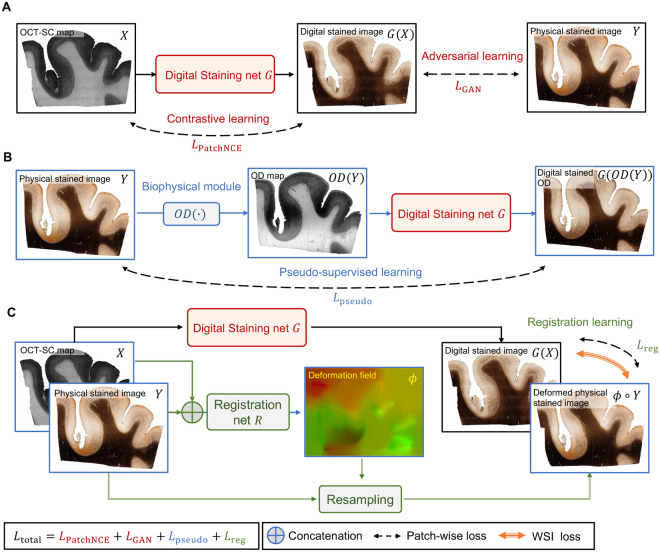
The training framework of our DS neural network model. (**A**) The backbone of the DS network *G* is built on the CUT framework, which combines contrastive learning and adversarial learning. The input is a 2D OCT-SC map *X* and the output is a digitally stained image *G*(*X*) that is compared with a PS image *Y* from an adjacent slice. (**B**) Auxiliary pseudo-supervised learning task. The biophysical module computes the optical density *OD*(*Y*) of the PS image *Y*, which is fed as an input to *G*. The digitally stained OD image *G*(*OD*(*Y*)) is compared with the original PS image *Y* during training. (**C**) Auxiliary unsupervised cross-modality image registration task. We alternate between optimizing *G* and a registration network *R* under different image scales. We fix *R* while updating *G*, which provides more informative supervision for *R* in the next iteration. We use patch-wise losses for training *G*, and whole slide image (WSI) losses for training *R*.

The training framework of our DS network consists of several novel learning components, as shown in Fig. 2. Based on the CUT framework as the backbone (32), the DS model uses a mix of adversarial loss and contrastive loss in the unpaired image setting, as shown in Fig. 2A. The adversarial learning measures the perceptual similarity of the generated DS images and the PS images. It tries to reduce the gap between the high-dimensional distributions of the DS and PS images such that the generated DS images are perceptually indistinguishable from the PS images. The contrastive loss uses self-supervised patch-wise learning to ensure structural consistency between the OCT-SC and DS images. It maximizes mutual information and provides self-guidance for content preservation. The combination of contrastive loss and adversarial loss enables high-quality DS images that preserve the content and structures of the OCT-SC images.

To improve upon the unsupervised CUT framework, we propose a semi-supervised learning method. Our method leverages augmented pseudo pairs generated by a biophysical model and registered cross-modality image pairs that are dynamically adjusted by a learnable registration network. The intuition is that using additional auxiliary supervision enhances the learnability, efficiency and accuracy of the model compared to unsupervised learning. Crucially, our semi-supervised method does not require any exact paired PS and OCT-SC images during training.

In Fig. 2B, we introduce the pseudo-supervised learning auxiliary task to enhance the unpaired image translation for DS of OCT-SC images. We first compute the OD maps from the PS images and then utilize the OD - PS image pairs to train the DS model in a pseudo-supervised manner. This approach proves effective because the OD image exhibits similar image contrast and feature distribution as the OCT-SC across various cortical regions. Additionally, the OCT-SC demonstrates an approximate linear relationship with the OD of the Gallyas silver stain (21). Furthermore, since the OD map is naturally pixel-aligned with the PS image, it facilitates supervised learning and provides additional semi-supervision and alignment constraints for the main DS model. However, the inherent disparities in image features and intensity value distributions between the OD map and the OCT-SC image result in a domain gap, which limits the accuracy of the trained DS model when relying solely on this auxiliary task. Our insight is that when this task is combined with the adversarial learning component in the CUT backbone, it enables domain adaptation similar to the domain-adversarial training framework (33). The performance on the OCT-SC image is ensured by penalizing the perceptual differences between the DS images generated from the OCT-SC image and the OD map using the adversarial loss. By leveraging both the pseudo-supervised learning and adversarial learning components, we effectively bridge the domain gap and improve the accuracy of the DS model for OCT-SC image translation.

In Fig. 2C, we illustrate the second auxiliary task for aligning the PS image, the OCT-SC image, and the DS image using a registration network. This registration module undergoes two training stages: pre-training and fine-tuning. During the pre-training stage, the registration module operates on the WSI scale. It predicts a deformation field that indicates the pixel-wise displacement vectors required for non-rigid transformation. To facilitate cross-modal self-supervised registration, we utilize the OD map as a surrogate for the OCT-SC image and learn a deformation field between the OD map and the input OCT-SC image. This result is used as an initial estimate for the deformation between the PS image and the matching OCT-SC image. By leveraging our biophysical model, we bootstrap the challenging self-supervised cross-modality image registration problem in this pre-training stage. The subsequent fine-tuning of the registration model aims to provide pixel-wise weak-supervision for the DS model. In this stage, we employ an alternate training approach that involves collaborative learning between the DS model and the registration model. When the DS model is fixed, the registration model is trained at the WSI scale to address global geometry correction. When the registration model is fixed, the DS model is trained at the image patch scale to provide sufficient samples for local translation learning. This unsupervised cross-modality image registration module enables the DS model to learn improved local color tone mapping from unaligned imaging modalities without the need for explicit supervision.

Overall, our DS framework augments unpaired image translation with pseudo supervised learning and unsupervised cross-modality image registration. The total loss function used for training is the weighted sum of the four objectives derived from the main image translation task and two auxiliary tasks. Our method achieves superior performance over other baseline methods, including CycleGAN, CUT and FastCUT in terms of DS quality and accuracy, as shown in Supplementary Materials (SM) Section 1, Section 2, Fig. S1 and Fig. S3. Additional details about the network structure, training procedures and quantitative evaluations are described in Methods and SM Section 3, 4, 9 and 10.

### Digital staining enhances mesoscopic brain structures and provides high staining uniformity

We present the ability of our DS technique to preserve the mesoscopic brain structures and achieve uniform staining of cerebral cortex sections from post-mortem human brains. We use two groups of PS imaging results as comparative references: one group consists of WSIs of well-stained sections, and the other group consists of WSIs of less-ideally-stained sections.

**Fig. 3.**
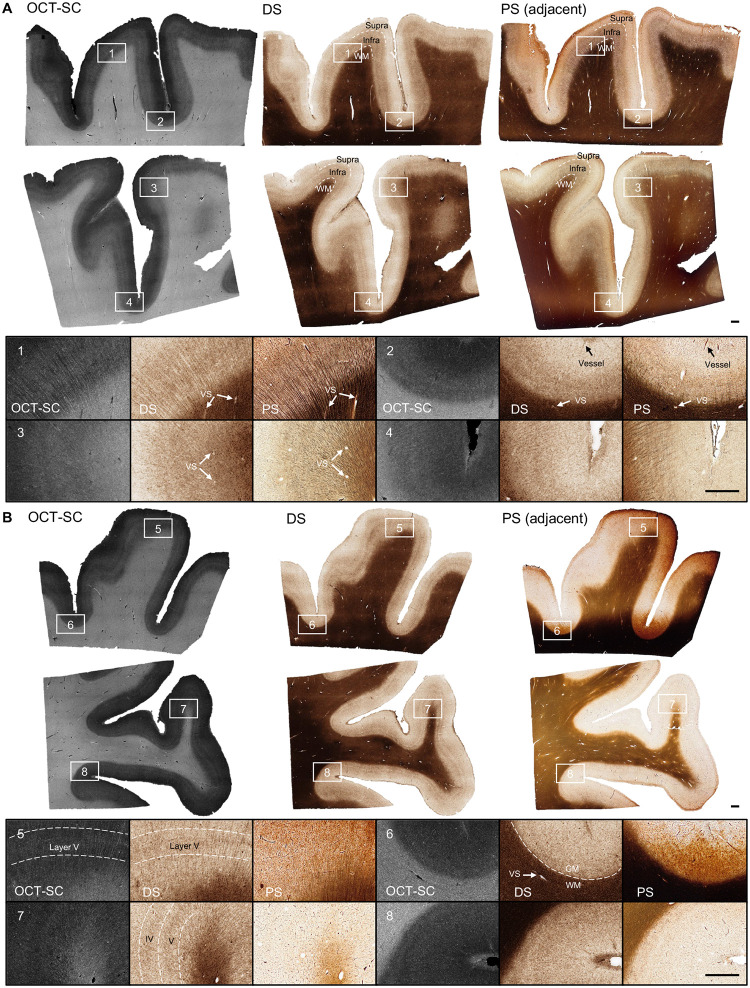
DS results on OCT-SC of tissue slices and comparisons with PS images. Cases include (**A**) ideal staining samples; (**B**) non-uniform staining and understanding samples. ROI 1, 3, 5, 7 are gyral crest regions and 2, 4, 6, 8 are sulcal fundus regions.VS: “vessel space”. Scale bars are 1 mm.

In Fig. 3A, we present the OCT-SC, DS, and well-stained PS images of adjacent sections from the human cerebral cortex, arranged from left to right. The DS images show that our technique can accurately capture various brain structures that match the PS images, such as cortical layers, myelin fibers, and vessel blobs. The DS and PS images share similar contrast, with white matter (WM) regions appearing as dark brown or black and gray matter (GM) regions appearing as white, while the OCT-SC image has the opposite contrast. Within the gray matter, the infra layers also appear to be darker than supra layers, consistent with the PS images. These correspondence in mesoscale structures validate that our DS model can reliably and accurately learn this general inverse mapping between OCT-SC and PS images.

In the zoom-in regions, we present the images on different types of cortex regions, including gyral crest regions marked as 1 and 3 and sulcal fundus regions marked as 2 and 4, from the three modalities: OCT-SC, DS and PS. In region 1, the structures of radial myelin fiber bundles at scales of about 10–20 μm are shown as dark brown tubular features in both DS and PS images, especially in the GM region. By comparing OCT-SC and DS images, we can see that the image content is consistent, which indicates that the ability of resolving fine features is primarily limited by the input OCT-SC data. Despite the limitations of resolution and speckle noise in the OCT data, the orientation of fiber bundle traces and the intensity distribution according to cortical layers can still be discerned in the DS results. Similar patterns are also evident in zoom-in regions 3 and 4, where the local intensity variation is visible in the GM regions, although the fiber bundles are less distinct in OCT-SC and DS images than in the PS images. In region 2, the supra cortical layers (I-III), infra layers (IV, V, VI) and WM are clearly distinguished by the white, light brown, and dark brown bands, respectively. The black line structure near the top of the PS image indicates smaller vessels, which are also visible in the DS image at the same locations. The zoom-in regions 1, 2 and 3 in PS show small white blob or tubeness features especially in the WM regions. In PS, these white blobs represent the empty space previously occupied by vessels which are lost due to slicing and washing steps during staining. In contrast, the white blobs in DS images primarily represent the space within vascular walls and perivascular space which appear smaller since no slicing or physical staining is performed on OCT-SC images. Those features are generally referred to as VS (“vessel space”) in Fig. 3. These visualizations demonstrate that our DS model can faithfully reveal ~20 μm scale brain structures.

A major advantage of DS over PS is stain uniformity. To demonstrate this, we present three types of images in Fig. 3B from the less-ideal PS group that comprises most of our PS data. One inherent limitation of traditional histological staining is the variability across different sample regions and experiments. Despite our careful sample preparation and staining procedures, the staining result is influenced by many confounding factors of the chemical reaction and uniformity of the staining quality is challenging to ensure. In Fig. 3B, the rightmost column of the first row shows a PS example with over- and non-uniform staining (in particular along the vertical directions); the second row shows a PS example with under-staining.

We select two gyral crest regions (marked as 5 and 7) and two sulcal fundus regions (marked as 6 and 8) to provide in-depth analysis. The PS images in regions 5 and 6 are over-stained, while the PS images in regions 7 and 8 are under-stained. In region 5, the DS and OCT-SC images show clear ridges corresponding to cortical layer V, but the PS image shows a dark brown shade due to over-staining. In region 6, which is a sulcal fundus region with less visible cortical layers, the DS image shows a clear boundary between WM and GM regions, but the PS image shows an ambiguous boundary. Small vessel blobs are also more visible in the DS image than in the PS image. In region 7, which is a gyral crest region, the DS image shows dark ridge features corresponding to cortical layer IV and V, but the PS image does not show these features due to under-staining. Additional examples are shown in SM Section 5 and Fig. S4.

The superior stain uniformity demonstrated by our DS results across different sections is enabled by the OCT-SC that extracts a normalized quantity based on a physics model that reflects the intrinsic property of the brain tissue. This stain uniformity will be a great advantage during anatomical and pathological evaluations. A limitation of our current OCT-SC curve fitting model is that it reduces the spatial resolution (lateral: 6 μm raw OCT measurement, 12 μm fitted SC map; axial: 6 μm raw OCT measurement, 150 μm fitted SC map), which limits the ability to resolve fine fiber structures.

### Digital staining enables reliable cortical layer differentiation and layer thickness quantification

We demonstrate the capability of DS-OCT to reliably distinguish cortical layers with comparable or even better sensitivity than PS, thanks to the uniform DS quality as discussed before. We identify cortical layers IV, V and VI by the displayed fiber density (36, 37), since these layers are more prominent than layers I, II and III in most of our samples. We provide additional examples of DS layer visualization and compare them with well-stained and less-ideal stained PS samples in SM Section 6 and Fig. S5. We also show how the layer thickness can be consistently quantified in our DS images.

**Fig. 4.**
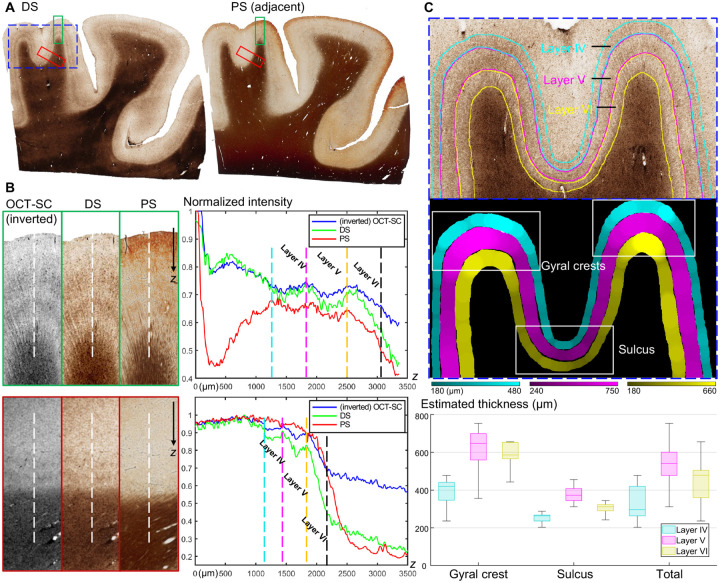
Comparisons results of layer differentiation and thickness estimation in DS results. (**A**) The DS and PS WSIs from a cortex tissue section. (**B**) Zoom-in ROIs of inverted OCT-SC, DS and PS modalities marked in green and red boxes in (A) and normalized intensity profiles aggregates along white dotted lines. (**C**) Manually annotated layers IV/V/VI labeled in three colors and estimated local thickness. Statistics of thickness are visualized in box plot and grouped by gyral crest and sulcus regions. ROI is the zoom-in of the dotted blue box from (A).

Figure 4A shows the WSIs of the DS result and the reference PS of an adjacent brain slice. The DS image clearly reveals the curved double-band structures above the WM region, which are stained in dark brown. These features indicate higher myelin fiber density that are characteristic in cortical layer IV and V (37). Consistent image contrast variations for the laminar structures are observed in the DS result. In contrast, the double-band structures are less visible around some of the gyral regions and the contrast is less distinct in the PS image. Figure 4B shows zoom-ins from a gyral crest region and a sulcus region of the three modalities, corresponding to the regions marked by the green box and red box in Fig. 4A respectively. The OCT-SC and DS images have a strong correlation in their intensity variations. The DS image consistently shows the double-band features in the GM region, while the PS image often fails to reveal them due to over- or under-staining.

Next, we demonstrate the improved contrast between cortical layers in DS by plotting the average intensity (across the three color channels) along the white dotted lines in Fig. 4B. The right panel shows the normalized profiles over a 3.5-mm depth range, where blue, green and red represent OCT-SC, DS and PS modalities, respectively. We manually marked the boundaries of layer IV, V and VI with dotted vertical lines in four different colors. In both gyrus and sulcus regions, the DS profiles show the highest contrast (measured by the difference between the maximum and minimum values) in layer IV and V among the three modalities, which facilitates identifying the layer boundaries. When comparing OCT-SC and PS with DS, the DS enhances the intensity variations at the boundary between layer IV and V. This reduces any confusion when distinguishing between these two layers. Comparing the profiles between OCT-SC and DS in different layers suggests that our DS model works beyond our approximate linear biophysical model (21) and increases the local contrast by a nonlinear mapping function expressed by our neural network.

In Fig. 4C, we further demonstrate straightforward segmentation and thickness quantification of cortical layers IV, V and VI using our DS result (see details in Methods), which can provide valuable information for many neuropathological studies (17, 38, 39). The top panel shows the zoom-in region of the dotted blue box in Fig. 4A, where we manually labeled the boundaries of the three cortical layers. We estimated the layer thicknesses from the binary mask obtained from cortical layer segmentation using an algorithm from our previous work (17). We chose two gyral crest regions and a sulcus region indicated by the white boxes in the binary mask image. The bottom panel displays the box plot of the local layer thickness statistics in gyrus and sulcus regions. We observed a similar pattern of variation in layer thickness for layer IV, V and VI in the sulcus, gyrus and the entire cortical regions. The median local thickness of layer IV, V and VI were 300 μm, 540 μm and 480 μm respectively. We also observed a significant reduction in layer thickness in all three layers in the sulcus regions compared to the gyrus regions, in agreement with the literature (40, 41). The median thickness of layer IV, V and VI were 410 μm, 630 μm and 580 μm respectively in the gyrus regions, and were 250 μm, 370 μm and 310 μm respectively in the sulcus regions.

### Volumetric digital staining on cubic centimeter-scale brain tissue

Next, we showcase volumetric staining on cubic centimeter-scale brain tissue enabled by our technique that combines S-OCT and DS. Our technique significantly reduces tissue distortion and misalignment during the 3D reconstruction process suffered by the traditional 3D pathology technique. We demonstrate 3D DS on a 4 cm × 5 cm × 1.2 cm brain tissue block that was not used for training our DS model. We show that our method can preserve the intricate 3D brain structures in both GM and WM regions. Moreover, we visualize the 3D vessel network in the WM.

**Fig. 5.**
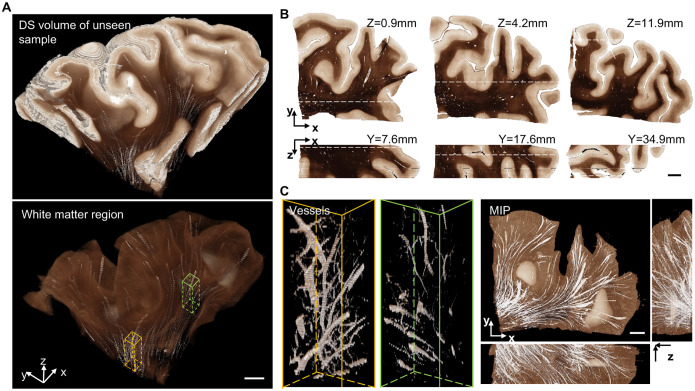
3D visualization and cross-sections views of the DS results on a large unseen tissue block. (**A**) The DS output images are stacked along the z-axis to render the whole digitally stained volume as well as segmented WM regions. (**B**) Orthogonal cross-sectional views of the DS volume. (**C**) Two zoom-in regions of vessel structures in yellow and green boxes from (A) are shown on the left. Three orthogonal maximum intensity projections (MIP) of the DS volume are shown on the right. All scale bars are 5 mm.

In Fig. 5A, we present a 3D visualization of the DS output on the whole tissue block in the top panel. The DS model takes as input a z-stack of around a hundred slices of OCT-SC images. Each OCT-SC slice, which has a size of 4 cm × 5 cm, is processed separately and fed to the DS model. The DS output images are then directly stacked along the z-axis to create the digitally stained volume. Consistent with the 2D results, the 3D DS volume generates white and dark-brown colors that correspond to GM and WM regions respectively. We can also observe a smooth transition of these GM and WM boundaries along the z direction, which reflects the preservation of 3D geometries of the brain structures. In Fig. 5B, we display several orthogonal cross-sectional views of the DS volume. The overall color tone and contrast variations match with the 2D results in Fig. 3. Small white blobs and tubes within the WM region indicate the vessel space. These results are consistent with 2D DS results that have been verified with PS references, and partly confirm the generalization ability of our DS model on unseen large-scale brain samples. Moreover, the X-Z cross section also shows several continuous features along the depth, such as intricate brain folding structures, double-band cortical layers, and small tubular vessels. This again illustrates the 3D geometry preservation feature of our DS technique.

To further illustrate the ability of our DS technique to preserve the 3D geometry of mesoscale brain structures, we present a 3D visualization of a centimeter-scale network of vessel space which is not visible in 2D PS images. Besides the GM and WM contrast, our DS volume also shows several continuous white tubular structures corresponding to blood vessels in the top panel of Fig. 5A. In the bottom panel of Fig. 5A, we show the segmented DS volume displaying only the WM region, where the white tubular structures are more prominent and not masked by the GM. In Fig. 5C, we highlight two regions in yellow and green boxes. The vessel spaces in those regions are rendered with more transparency and reveal the branching and connectivity of the vessel network. On the right panel of Fig. 5C, three orthogonal maximum intensity projections (MIP) of the DS volume further demonstrate the preservation of the 3D vessel structures. We note that the axial continuity of our DS volume is currently limited by the axial resolution (150 μm) imposed by our SC fitting model, which we aim to improve in the future. Being able to image brain samples as large as 4 × 4 × 1 cm^3^ (34), we can easily extend the aforementioned analysis to large brain areas with uniform and enhanced contrasts, which could greatly improve the throughput of brain anatomy study.

### Generalization to unseen anatomical regions

**Fig. 6.**
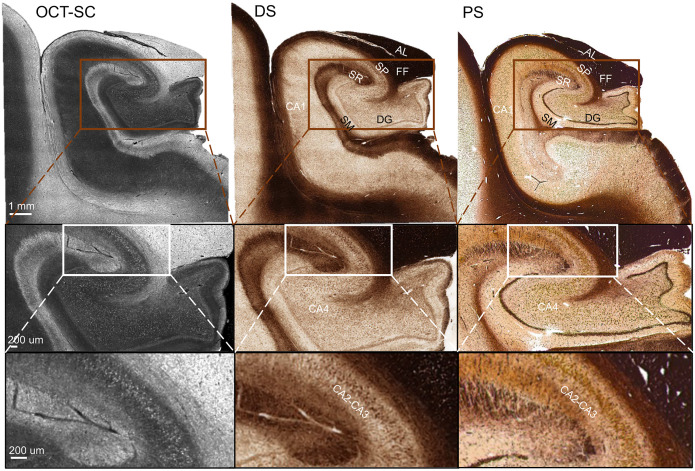
DS-OCT generalization performance on a hippocampus tissue slice. Examples of OCT-SC, DS and PS images (of adjacent sections) on one sample from the Hippocampus region are shown. SP: Stratum Pyramidale; AL: Alveus; FF: Fimbria Fomix; SR: Stratum Radiatum; SM: Stratum Moleculare; DG: Dentate Gyrus; CA: *Cornu Ammonis*.

To further demonstrate the generalization capability of our trained DS model, we conducted a pilot study on different anatomical regions that were imaged by a different S-OCT setup not seen during training. We used the same fitting model to generate the OCT-SC image in Fig. 6, which shows a sample from the hippocampus region acquired by a different S-OCT setup. Since our SC fitting model extracts an intrinsic tissue property and is relatively insensitive to variations in hardware platforms and sample conditions, it ensures the robustness of our DS method. The DS image is inferred by directly inputting the OCT-SC to the previously trained model. Figure 6 shows the OCT-SC and DS images, and the reference PS image of an adjacent section from left to right. We roughly aligned the field of views of the DS and PS images using a rigid transformation. On a large scale, the DS process successfully transforms the image contrast to match the anatomical structures found in the PS image. By comparing with the anatomy of hippocampus (42), we can identify the alveus (AL) and/or fimbria fomix (FF) layer at the top, the stratum pyramidale (SP) layer beneath them, and the stratum radiatum (SR), stratum moleculare (SM) and the dentate gyrus (DG) layers that encase the *Cornu Ammonis* areas (CA1–CA4) of dense neurons. Importantly, in CA1–CA4 areas, we found bright spots in OCT-SC images, which are transformed to brown spots in the DS images. These structures correlate strongly with the brown spots seen in the PS image and are likely individual neuron somas. More examples of generalization results can be found in SM Section 7 and Fig. S6.

Such generalization agrees with our previous work that discovered a universal correlation between optical scattering and myelin density across the human brain (21). This suggests that a DS-OCT model, even if trained on limited regions of the human brain, may be effectively employed in other unseen regions. This significantly decreases the training effort compared to those that rely on transfer learning.

## Discussion

In summary, we developed a novel semi-supervised learning technique for DS of OCT images for large-scale volumetric visualization and analysis on human brain tissue samples. Our technique works by integrating label-free S-OCT imaging and an advanced deep learning DS model. The S-OCT enables imaging of cubic centimeter-scale brain tissues and preserves complex 3D tissue geometry across sections. Our semi-supervised learning method bypasses the need for paired unstained and stained images and can achieve high-quality DS using a limited amount of weakly paired image data for model training. Our deep learning model is built on an unsupervised CUT model backbone, which is augmented with two auxiliary tasks. The pseudo-supervised module reduces the data requirement for model training by exploiting the correlation between the OCT-SC and the OD of Gallyas silver stain. The unsupervised cross-modality image registration module exploits the structural information between the adjacent tissue sections. By working with a fitted tissue property, namely the SC, from the raw OCT measurement as the input to the deep learning model, it greatly enhances the uniformity and generalizability of the DS results. This is highlighted by our volumetric DS result on cubic centimeter-scale brain tissue block and on unseen anatomical regions from different OCT systems. We believe our OCT DS technique is a promising solution for large-scale human brain imaging for comprehension characterization of brain structure across scales.

We envision that our deep learning framework holds great potential for a wide range of applications in the field of DS. There is a growing demand for exploring semi-supervised learning approaches to effectively harness the wealth of information contained in unpaired or weakly paired biomedical images. Obtaining pairs of images with labels and without labels can be a challenge in many biomedical contexts. However, it is often easier to obtain images of samples with slight distortions or adjacent sections. To leverage these types of datasets, our method leveraged a novel inverse mapping technique, going from stained images to label-free modalities, and generated pairs of images that were pixel-aligned to serve as augmented supervision. Furthermore, we introduced a novel cross-modality registration algorithm to correct for sample distortions and account for the complex geometries of the samples. As a result, our enhanced semi-supervised learning framework facilitates more straightforward training on datasets that may be naturally acquired from routine staining experiments, even when those datasets are only weakly paired. In essence, incorporating semi-supervised methods can significantly enhance the efficiency of the “data collection-training-validation” cycle in the development of digital staining models.

We discuss some of the main limitations that affect the quality of S-OCT images and the DS method based on them. The first limitation is the data processing pipeline of OCT imaging. Coherent scattering results in speckle noise, which manifests as randomly distributed fine-grained dark or white spots in OCT and the fitted SC images. These speckle artifacts do not necessarily correspond to the actual cortical structures in PS images, as shown in Fig. 3 and SM Fig. S4. Consequently, visualizing and digitally staining small vessels, capillaries, and fine axonal fiber structures become challenging. Moreover, the current resolution of our OCT-SC data is insufficient to resolve delicate structures like single neurons. To address this limitation, a possible future direction is to optimize the processing pipeline of OCT-SC with deep learning techniques to achieve higher imaging quality. For example, self-supervised learning algorithms for speckle suppression can be developed by utilizing a customized blind-spot denoising network and a speckle statistics model (43). Enhancing the resolution of SC can be explored by employing a deep learning model similar to (44) to learn a more accurate and robust fitting model without the need for local-averaging. These improvements can increase the robustness and resolution of our method, enabling us to capture finer neuronal structures. The second limitation pertains to stitching artifacts that cannot be fully corrected in our current DS model, thereby affecting the quality of WSI image, as observed in Fig. 3, 4 and SM Fig. S6. To address this issue, it may be possible to incorporate a structural prior constraint into our DS training framework, which will potentially yield better correction of these artifacts. The last limitation involves the imperfect registration component in our DS model. The fitting depth range we utilized for SC (150 μm) is larger than the physical sectioning thickness of PS images (50 μm). Furthermore, during staining experiments, sample destruction may occur, introducing imaging content mismatch. However, our registration learning only corrects for global-scale geometric distortion between adjacent sections and does not account for potential content mismatch between weakly-paired images. Consequently, the registration process fails to generate pixel-aligned image data, as seen in SM Fig. S2. To tackle this issue, further improvements to the deep learning framework may consider methods to address content mismatch.

It is worth noting that our training and testing images comprise a mix of normal control and neurodegenerative human brain samples, which hinders the model’s ability to learn the distinctions between normal and diseased brain images. To expand our work towards distinguishing between normal and diseased cases, one needs to acquire images from a larger set of brain samples for both conditions. Additionally, we plan to incorporate multi-modality input, such as polarization information, into our DS model to increase the imaging sensitivity to birefringence structures, including myelin fibers (17, 19). Another promising modality we aim to integrate with the S-OCT is two photon microscopy, which allows imaging of small vessels and myelin fibers based on autofluorescence contrast with reduced noise and improved resolution (34).

## Materials and Methods

### Serial-sectioning OCT (S-OCT)

The S-OCT microscope was described previously (34). We used a swept light source (AxsunTech) with 100 kHz swept rate, a central wavelength of 1310 nm, and a spectral full width half maximum (FWHM) of 110nm, yielding an axial resolution of 5.6 μm in brain tissue (n=1.4). We used a free-space interferometer and quarter wave plate (QWP) to illuminate the sample with circularly polarized light, and used two balanced detectors for measuring orthogonally polarized reflection light. A variable retarder (VR) placed in the sample arm was used to compensate for the system birefringence and to recover precise measurement of sample birefringence. To sample the spectrum in even-k space, we input the k-clock of the light source into a high-speed digitizer (ATS9350, AlazarTech), afterwards real-time FFT was carried out using a Graphic Processing Unit (RTX4000, NVIDIA), and the spatial-domain data was trimmed to only save the first 1 mm depth. The post-objective power was measured to be 3.7 mW, achieving a 95dB SNR in both polarization channels. We used 1×1 mm^2^ FOV with 3 μm lateral step size and 30% overlap between tiles. The sample was mounted on XYZ motorized stages which translated the sample to image the whole surface as well as between the vibratome and objective. After block-face imaging, a custom vibratome cut off the 50 μm slices with 0.3mm/s cutting speed and 3000 rotations per minute (RPM) blade vibrating frequency.

### Optical properties estimation with S-OCT

Tissue optical properties were extracted by following a previously established procedure to analyze the reflectance intensities of OCT using a nonlinear fitting method (11, 12). To summarize, spatial parametrization is first applied to the confocal parameter across a 3D OCT image to constrain and reduce the degrees of freedom in the nonlinear coefficient fitting problem, resulting in improved confidence in the estimated optical properties of the sample. Afterwards, a nonlinear least-squares solver is used to estimate the back-scattering and scattering coefficients from the nonlinear reflectance-vs-depth over about 150 μm depth. All curve fitting was performed in MATLAB. After extracting the optical properties for each image tile, the tiles were stitched using the coordinates generated during the volumetric reconstruction with ImageJ software (45).

### Sample preparation

For the training phase, we used a set of 15 samples obtained from the Boston University Alzheimer’s Disease Research Center brain bank. These samples consisted of five cases with stage VI Alzhemer’s disease (AD), five cases with stage III and IV Chronic Traumatic Encephalopathy (CTE), and five age-matched normal control cases. To ensure representation across the thickness of the tissue, we selected one slice per millimeter for this study.

For the pilot generalization study, we used OCT data from five samples obtained from two human brains. These samples were collected at the Massachusetts General Hospital Autopsy Suite and encompassed various brain regions, including the cerebellum, hippocampus, somatosensory cortex, superior frontal cortex, and middle temporal area 21. The subjects from whom these samples were obtained had no history of neurological deficits and had a mean age of 53.5 ± 12.0 years, with one male and one female.

All samples were fixed by immersion in 10% formalin for at least two months. The post-mortem interval did not exceed 24 hours. Prior to imaging, the samples were washed in 1X phosphate buffered saline for a month to remove residual fixation agents and then embedded in 4.5% agarose for tissue support (46). During embedding, the brain blocks were warmed to 65 °C to allow sufficient penetration of agarose into the deep sulcus. During imaging, the brain tissue blocks were mounted in a water bath filled with Deionized (DI) water. The DI water was changed every day to remove the debris from cutting that could degrade the OCT image quality. Following data collection, the tissue slices were stored in 1X PBS with an antibacterial agent (sodium azide) at a temperature of 4 °C. To maintain the sequence of the slices, each slice was stored in an individual glass vial.

### Gallyas silver staining and imaging

A total of 35 brain slices were obtained from 15 samples for our study. To ensure anatomical diversity, at least two slices were taken per sample, with each slice being separated in depth by 1 mm. These slices had a thickness of 50 μm and were mounted onto gelatin-coated slides. Gallyas staining protocol, as described by Pistorio (2), was then employed to process the samples. Modifications were made to the impregnation and bleaching time to accommodate the increased thickness of the samples.

Following the staining process, the samples were captured in brightfield mode using a 10 × objective (NA=0.4) and an RGB camera. We utilized the VS-120 slide scanner designed for 75 × 25 mm^2^ slides for this purpose. The exposure time was set at 1.73 ms, and the pixel size was 0.7 μm with a 1 × 1 mm^2^ FOV. For wider samples, imaging was conducted using the BZ-X microscope under similar settings. The resulting images can be opened in Olympus Olyvia software and exported as TIFF images for further processing.

### Image processing

Our image dataset consists of two types of images: PS images from the slide scanner and OCT-SC images computed from S-OCT. We first inspected all the PS images visually and excluded the ones that had low staining quality or artifacts in the training dataset. We selected 9 out of 35 PS WSIs for training our DS model. The PS WSIs had different sizes depending on the tissue sample, but they were around the median scale of 36 mm × 48 mm with the pixel size of 1.9 μm. To generate the weakly-paired training dataset, we manually paired the PS images with the OCT-SC images that had the most similar appearance. Since the sectioning thickness (50 μm) of PS samples did not match the fitting thickness used for OCT-SC images (150 μm) and the depth information of PS samples was not recorded, we can only pair the PS with the closest adjacent OCT-SC image sample by qualitatively assessing the similarity of tissue features. We then downsampled the PS images using bicubic interpolation by a scale factor of 6.32 to match the 12 μm pixel size in OCT-SC images. We also cropped or padded the PS images to have the same image size as the corresponding OCT-SC images, which was around 3000 × 4000 pixels for each sample. We performed this procedure on all PS images when we compared them with the OCT-SC images side-by-side in our results.

The PS images undergo several preprocessing steps to minimize the effects of sample and staining variations before they are used for training. The preprocessing steps include background removal, intensity normalization and color transfer. The background removal eliminates the unwanted image artifacts in PS image and is done by interactive image segmenter in MATLAB. The intensity normalization adjusts the PS images to balance the varying illumination levels across different imaging experiments. The brightest pixel (I_r_, I_g_, I_b_) is used to estimate the illuminant color and the image is scaled by the constant (1/I_r_,1/I_g_,1/I_b_) for each color channel, followed by a range normalization to map the overall image value range to [0, 1]. The color transfer uses Reinhard method (47) to standardize the staining color variations among experiment, sample and imaging conditions given a target PS image with a relatively ideal staining as reference.

The OCT-SC images obtained from the fitting algorithm show some artifacts mainly in the background areas and near the sharp boundaries of the vessel regions, because the algorithm assumes a constant SC value for the 150 μm imaging thickness (11). To reduce the background noise and correct the over-smoothed values near the vessel edges, the OCT-SC images are further processed by several steps. First, the background is removed by applying a histogram-based thresholding method using the triangle algorithm (48), followed by a sequence of smoothing morphological operations such as erosion, small object removal and dilation. Next, the pixels with zero values in the masked image are identified as defective and are replaced by the local median. Then, the edges of the vessel regions are detected using a difference-of-Gaussian (DoG) filter and thresholding. Finally, the outlier regions with small values compared to the local maximum are segmented and combined with the edge mask. The combined mask is smoothed by similar morphological filters, and the values in the mask are replaced by the local maximum. The preprocessing pipeline is implemented in Python using the basic filters and morphological operators from scikit-image package (48).

To generate the training image dataset, we used PyTorch to create a parallel processing module that can split the WSIs of different image sizes into smaller patches during training on the fly. This allows us to dynamically update the intermediate image tensors that can be input to different parts of deep learning models to train at different image scales. The WSIs dataset with different sizes can then be directly handled by a custom data loader for standard-size tensor operation. We first pad the WSI to the size of multiple integers of patch size, and then use the tensor unfolding method in PyTorch to cut the image tensor using a sliding window into smaller tensors stacked in the batch dimension. The inverse stitching operation is done similarly using the tensor folding methods.

For creating a 3D visualization of the DS images that show the volumetric digital staining results, we change the white-color background of the DS images to black, so that only the sample region is visible. This is done by converting the DS color images to grayscale and applying a triangle method threshold to select the foreground pixels. Then, a morphology smoothing operation is performed to remove any noise or artifacts. To extract the WM masks from the DS grayscale images for highlighting the WM regions in the sample, we use a histogram thresholding method based on the minimum method (48) and apply another morphology smoothing operation. The pixels that are not part of the WM masks are set to zero, and the resulting images are stacked in a volume for 3D visualization. The 3D viewer in ImageJ (45) is used to display the volume. More details on the image processing procedures are provided in SM Section 8 and Fig. S7.

### Semi-supervised deep learning framework

The proposed framework combines generative adversarial learning, contrastive learning, pseudo-supervised learning based on self-generated image pairs based on a biophysical model, and unsupervised cross-modality image registration.

We denote the OCT-SC images as *X* and the PS images as *Y*. The main framework consists of a DS network *G* and a registration network *R*. The DS network *G* transforms grayscale OCT-SC images *X* into color images that resemble the color and contrast of PS images Y. The registration network R takes pairs of unaligned images *X* and *Y* as input and outputs a deformation field *ϕ* = *R*(*X*, *Y*) that can be applied to resample and register *Y* to *X*. We use an auxiliary discriminator network *D* to enforce structural similarity between the output DS and reference PS images by adversarial learning. We also apply contrastive learning to ensure structural consistency between the input OCT-SC and output DS images using a fully connected network *f*.

Our framework operates on two different image scales: WSI scale (denoted by upper case letters) and image patch scale (denoted by lower case letters). *R* is trained on WSIs, which have a size of about 3000 × 4000 pixels. *G, D, f* are trained on image patches, which have a size of 512 × 512 pixels. We design an efficient image processing module to either split (*X, Y*) into patches (*x, y*) or stitch patches back to WSIs, as detailed in the Image Processing section. The CUT framework (32) is used to jointly train the networks *G, D*, and *f* during the training phase. Additionally, *G* undergoes a pseudo-supervised training scheme and an alternating training process with *R*, which are explained below.

The objective of the adversarial learning module is to enhance the perceptual similarity between the DS output *G*(*x*, *y*) and the target modality PS images *y*. This is achieved by using an auxiliary discriminator *D*. The role of *D* is to learn to differentiate between the desired modality y and the generated images *G*(*x*). During the training of *D*, the PS images *y* are assigned the label 1, indicating that they are “true” images. On the other hand, the generated images *G*(*x*) are assigned the label 0, indicating that they are “false” images. The least-squares generative adversarial network (GAN) loss *L*_GAN_(*D*) is employed to measure the extent to which *D*’s outputs align with the binary labels assigned to both *y* and *G*(*x*). This loss function is minimized when *D* becomes proficient at distinguishing between *y* and *G*(*x*). Conversely, when training *G*, the *L*_GAN_(*G*) loss is utilized to promote the fidelity of the generated images *G*(*x*). Minimizing this loss prompts *G* to effectively deceive the discriminator *D*. The training process alternates between two steps: first, *G* is fixed while *D* is updated using the *L*_GAN_(*D*) loss, and then *D* is fixed while *G* is updated using the *L*_GAN_(*G*)loss:

(1) $$L_{\text{GAN}}(D)=E_{y}\left[{\left(D(y)-1\right)}^{2}\right]+E_{x}\left[D^{2}(G(x))\right]$$

(2) $$L_{\text{GAN}}(G)=E_{x}\left[{\left(D(G(x))-1\right)}^{2}\right]$$

The contrastive learning module ensures that the image structures and content present in x is preserved when it is translated to *G*(*x*) We implement *G* with a ResNet model and treats the first half of the ResNet layers as the encoder and the remaining layers as the decoder. The encoder G_enc_ transforms images from both domains into a common latent space, and the decoder G_dec_ generates translated images from latent vectors. To formulate the multi-layer patch-wise contrastive loss, we adopt the approach in (32) to sample the encoded feature maps from both *x* and *G*(*x*). Each layer and spatial location in the feature map stack corresponds to a patch of the input image that covers the corresponding receptive field. We extract multiple layers of the encoded feature maps, randomly sample the spatial locations and apply a fully connected network f to obtain a stack of latent features ${\hat{z}}_{s,l}=f\left(G_{\text{enc}}^{s,l}(x)\right)$, where *s* is the spatial index within [1, *S*] and l is the selected layer within [1, *L*]. We do the same processing on image $G(x):{\hat{z}}_{s,l}=f\left(G_{\text{enc}}^{s,l}(G(x))\right)$ Then we compute a PatchNCE loss using a cross-entropy contrastive loss:

(3) $$L_{\text{PatchNCE }}(G,f,x)=E_{x}\overset{L}{\underset{l=1}{\sum }}\overset{S}{\underset{s=1}{\sum }}\log\left(\frac{\exp\left(z_{s,l}\cdot {\hat{z}}_{s,l}\right)}{\sum_{t=1}^{s}\exp\left(z_{s,l}\cdot {\hat{z}}_{t,l}\right)}\right)$$

This loss function encourages the latent representations of image patches from *x* and *G*(*x*) that belong to the same spatial location to have similar content to be close in the feature space, while pushing away the representations of patches that are uncorrelated or have different content. By this internal negative sampling scheme in the feature space, the model learns to contrast positive and negative pairs of patches based on their content similarity, which maximizes the mutual information between the input image *x* and the output image *G*(*x*) This provides a self-supervised signal for preserving the content of the image during the transformation.

The training procedure for pseudo-supervised learning is formulated as a pixel-wise loss function that minimizes the discrepancy between the digital stained OD images *G*(OD(*Y*)) and the physical Gallyas-silver stain (PS) images *Y*. This loss function aims to guide G to learn a mapping that translates images from the OD modality to the PS modality. By doing so, it provides a “proxy supervision” for learning the mapping from OCT-SC modality to the PS modality. To facilitate this training, we first compute the OD of image *Y* by

(4) $$\text{OD}(Y)=-\frac{1}{3}\underset{c=R,G,B}{\sum }\log_{10}Y_{c}$$

Subsequently, we extract patches OD(*y*) and *y* from the processed WSIs and employ an auxiliary pseudo-supervised loss, defined as:

(5) $$L_{\text{Pseudo }}(G)=E_{y}∥G(\text{OD}(y)-y{∥}_{1}$$

However, there exists a mismatch in the intensity values between *X* and OD(*Y*). This domain gap between the input modalities hinders the model’s direct generalization on *X* if it is solely trained on pairs of OD(*Y*) and *Y*. To address this issue, we first apply histogram equalization to the WSIs of OD(*Y*) and *X* before feeding them into *G*. This normalization step aims to align the distribution of intensity range. However, we found that this transformation alone is insufficient in mitigating the domain gap. As a result, this learning module is further combined with the adversarial learning module in the CUT backbone to mitigate the domain gap between OCT-SC and OD.

The cross-modality image registration module is trained in two stages. In the first stage, we pre-train the registration network *R* on WSIs of *X, Y* and OD(*Y*). The registration network *R* takes weakly-paired *X* and *Y* as input and predicts a deformation field *ϕ* = *R*(*X*,*Y*) that indicates the pixel-wise displacement vectors needed to perform non-rigid transformation. To formulate a cross-modal self-supervised registration loss $L_{reg}^{I}$, we use OD(*Y*) as a surrogate of *Y* and exploit its correlation with the input OCT-SC image *X*. By minimizing the difference between the registered OD(*Y*) and *X*, we indirectly learn the deformation between *Y* and *X*. This training is enabled by a differentiable resampling layer that performs image warping denoted by ∘. We also add a total variation (TV) regularization term to encourage the smoothness of the learned deformation field. The registration loss during this pre-training stage is computed at the WSI scale as follows:

(6) $$L_{\text{reg}}^{I}(R)=E_{X,Y}∥X-\phi \circ OD(Y){∥}_{1}+∥\phi {∥}_{TV}$$

where $∥\phi {∥}_{TV}$ is the total variation norm calculated as:

(7) $$∥\phi {∥}_{TV}=\underset{i,j}{\sum }\sqrt{{\left|\phi_{i+1}-\phi_{i,j}\right|}^{2}+{\left|\phi_{i,j+1}-\phi_{i,j}\right|}^{2}}$$

In the second fine-tuning stage, we train *R* and *G* in an alternating and collaborative manner. The purpose of fine-tuning *R* is to provide pixel-wise weak-supervision between the registered *Y* and the DS image *G*(*x*), which in turn helps to fine-tune the DS network *G*. Using the coarsely trained *G*, we can produce *G*(*x*) that has the same image modality as the PS image *Y* and use a pixel-wise loss function to perform training. We implement the following scheme for alternating training. When we fix *G*, we train *R* by comparing the registered PS image *Y* and the DS image *G*(*X*) at the WSI scale using the loss function

(8) $$L_{\text{reg}}^{II}(R)=E_{X,Y}∥G(X)-\phi \circ Y{∥}_{1}+∥\phi {∥}_{TV}$$

When we fix *R*, we crop the intermediate registered WSI *ϕ* ∘ *Y* into patches *ϕ*_y_ ∘ *Y* and train *G* at the patch scale by comparing the registered PS image patch and the DS image patch *G*(*x*) using the loss function

(9) $$L_{\text{reg}}^{II}(G)=E_{x,y}∥G(x)-\phi_{y}\circ y{∥}_{1}$$

Additional details about the deep learning framework and individual model architectures are provided in SM Section 9, 10 and Fig. S8, S9 and S10.

### Image analysis

The layer differentiation analysis is primarily performed using the open-source ImageJ software package. The line profiles are computed by selecting the rectangular region in the center region of interest (ROI) and aggregating the intensity value along the horizontal direction. Those profiles are then normalized to [0, 1] by their individual value range for visual comparisons. The cortical layer boundaries are manually annotated by identifying the local maxima and edges according to (36, 37). The layer segmentation on the larger ROI is performed by manual annotation on layer IV, V and VI. We used the built-in local thickness estimation function to generate the local thickness map and calculated the box plot for the thickness distribution using Matlab. Two Gyral crest ROIs and one Sulcus ROI are manually selected. Additional details about the analysis methods for the myelin fibers and vessel quantification are provided in SM Section 11 and Fig. S11.

## Data Availability

All data are available in the main text or the supplementary materials.
